# Grazing intensifies degradation of a Tibetan Plateau alpine meadow through plant–pest interaction

**DOI:** 10.1002/ece3.1537

**Published:** 2015-05-29

**Authors:** Hui Cao, Xinquan Zhao, Shiping Wang, Liang Zhao, Jichuang Duan, Zhenhua Zhang, Shidong Ge, Xiaoxue Zhu

**Affiliations:** 1 Key Laboratory of Adaptation and Evolution of Plateau Biota, Northwest Institute of Plateau Biology, Chinese Academy of SciencesXining, 810008, China; 2 Graduate University of Chinese Academy of SciencesBeijing, 100049, China; 3 Chengdu Institute of Biology, Chinese Academy of SciencesChengdu, 610041, China; 4 Key Laboratory of Alpine Ecology and Biodiversity, Institute of Tibetan Plateau Research, Chinese Academy of SciencesBeijing, 100101, China; 5 Binhai Research Institute in TianjinTianjin, 300457, China

**Keywords:** Alpine meadow, experimental warming, grassland caterpillar, grazing, *Gynaephora menyuanensis*, plant–pest interaction, Tibetan Plateau

## Abstract

Understanding the plant–pest interaction under warming with grazing conditions is critical to predict the response of alpine meadow to future climate change. We investigated the effects of experimental warming and grazing on the interaction between plants and the grassland caterpillar *Gynaephora menyuanensis* in an alpine meadow on the Tibetan Plateau in 2010 and 2011. Our results showed that grazing significantly increased nitrogen concentration in graminoids and sward openness with a lower sward height, sward coverage, and plant litter mass in the community. Grazing significantly increased *G. menyuanensis* body size and potential fecundity in 2010. The increases in female body size were about twofold greater than in males. In addition, grazing significantly increased *G. menyuanensis* density and its negative effects on aboveground biomass and graminoid coverage in 2011. We found that *G. menyuanensis* body size was significantly positively correlated with nitrogen concentration in graminoids but negatively correlated with plant litter mass. Even though warming did not significantly increased *G. menyuanensis* performance and the negative effects of *G. menyuanensis* on alpine meadow, the increases in *G. menyuanensis* growth rate and its negative effect on aboveground biomass under the warming with grazing treatment were significantly higher than those under the no warming with grazing treatment. The positive effects of grazing on *G. menyuanensis* performance and its damage were exacerbated by the warming treatment. Our results suggest that the fitness of *G. menyuanensis* would increase under future warming with grazing conditions, thereby posing a greater risk to alpine meadow and livestock production.

## Introduction

The Tibetan Plateau (TP) has experienced significant warming since the 1950s, exceeding the temperature increases observed for areas within the same latitude of the Northern Hemisphere (Liu and Chen 2000; Liu et al. 2009), and future warming on the TP is predicted to be greater than the global average (Rangwala et al. 2013). Alpine meadows, covering about 35% of the plateau area, comprise the representative vegetation on the TP. Livestock grazing is the dominant form of land use in the meadows (Zhao and Zhou 1999). Climate warming and grazing are two major drivers of environmental change in alpine meadows on the TP. The relatively robust literature are on the response of plant biomass, plant nutritive quality, species composition, and species diversity in alpine meadow communities to warming and grazing (Klein et al. 2004, 2007, 2008; Wang et al. 2012); however, less is known about the impacts of warming and grazing on plant–herbivore interactions (Li et al. 2011; Liu et al. 2011; Cease et al. 2012).

Grassland caterpillars, *Gynaephora* species (Lepidoptera: Lymantriidae), are major pests in the TP. *Gynaephora menyuanensis* is endemic to the northern TP and devours graminoid leaves in alpine meadows (Yan et al. 1995). During outbreaks, grassland caterpillars not only aggravate grassland degeneration for the reduction of graminoids, but also increase livestock mortality for shortages of fodder vegetation during winter (Zhang and Yuan 2013). Furthermore, domestic animals and wildlife may suffer from oral mucous membrane canker and broken-tongue disease as a result of feeding on cocoons of *G. menyuanensis* that remain in the meadows after outbreaks (Qiu et al. 2004). Preliminary investigations suggest that *G. menyuanensis* occurs across about 1.3 million ha of alpine meadow used for grazing and causes nearly US$1 million in herbage loss and chemical control costs annually (Ma 2002). Therefore, the performance of *G. menyuanensis* under warming with grazing conditions is critical to the health of alpine meadow and livestock under future climate change.

This study is to examine the effects of warming and grazing on the interaction between plants and *G. menyuanensis* through a controlled warming–grazing experiment. Our research objectives were to address the following two questions to estimate *G. menyuanensis* performance and its potential damage to alpine meadow under future climate change: (1) How does *G. menyuanensis* respond to the temperature and plant-mediated changes induced by experimental warming and grazing? (2) How does *G. menyuanensis* damage on aboveground biomass and graminoid coverage respond to experimental warming and grazing?

## Materials and Methods

### Study site

This study was conducted at the Haibei Alpine Meadow Ecosystem Research Station (latitude: 37°37′N, longitude: 101°12′E, elevation: 3200 m), which has a continental monsoon climate with severe, long winters and short, cool summers. The mean annual temperature and precipitation are 1.6°C and 562 mm, respectively, and over 80% of the precipitation falls during the summer monsoon season (Zhao and Zhou 1999). Mean temperature and total rainfall were 8.9 and 8.1°C and 412.6 and 307.8 mm during the growing seasons (May–September) in 2010 and 2011, respectively.

Graminoids, such as *Kobresia humilis* (CA Mey. ex Trautv.) Serg., *Carex scabrirostris* Kükenth, *Elymus nutans* Griseb, *Poa pratensis* L., and *Stipa aliena* Keng, dominate the plant community at the Haibei Alpine Meadow Ecosystem Research Station. Broadleaf weed species, including *Gentiana straminea* Maxim, *Potentilla nivea* L., *Potentilla anserina* L., *Saussurea superba* Anthony, and *Lancea tibetica* Hook. f. et Thomas, are present and can also be abundant at times.

### Experimental design and treatments

A two-way factorial design (warming and grazing) was used to generate four treatments: no warming with no grazing (NWNG), no warming with grazing (NWG), warming with no grazing (WNG), and warming with grazing (WG). In total, 16 plots (4 treatments × 4 replicates), each 3 m in diameter, were fully randomized throughout the study site.

A more detailed design of the controlled warming (i.e., free-air temperature enhancement system with infrared heaters) with grazing experiment was described previously by Wang et al. (2012). In brief, eight hexagonal arrays of MOR FTE (1000 W, 240 V; Mor Electric Heating Association, Comstock Park, MI) infrared heaters were deployed over the vegetation in the warming plots, and eight dummy arrays were deployed over the no warming plots in May 2006. The set-point differences between warming and no warming plots were 1.2/1.7 and 1.5/2.0°C (daytime/nighttime) in the summer and winter, respectively, which were controlled using a proportional-integral-derivative-outputs control system.

The grazing treatment conducted during 2006 to 2010 was described in detail by Wang et al. (2012). Two adult Tibetan sheep were fenced in each of the grazing plots on 7 July and 23 August 2010. The cumulative forage utilization rates were 55.5% for the NWG treatment and 57.7% for the WG treatment, respectively. The percentage of vegetation removed corresponded roughly to a moderate grazing/stocking rate for the study region. In 2011, there was no grazing during summer.

Cages for the grassland caterpillars (40 × 40 × 60 cm, length × width × height) were constructed of 40-mesh white nylon screen and installed in a similar position in each plot. To prevent caterpillars from escaping, steel base frames (42 × 42 × 7 cm, length × width × height) were driven into the soil, with 2-cm-deep and 2-cm-wide grooves to fix the cages. The effect of grazing was simulated by clipping in cages of grazing plots.

### Plant litter mass and nitrogen concentration of graminoids

Plant litter samples in all plots were collected from two 10 × 10 cm squares in August 2009, which were oven-dried at 80°C and weighed in the laboratory. Mature leaves of each graminoid species were collected, sun-dried in the field, and oven-dried at 60°C upon returning to the laboratory in August 2010. Total nitrogen (N) in mature leaves (an equivalent mixed sample) was assayed using an elemental analyzer (2400 II CHNS/O Elemental Analyzer; Perkin-Elmer, Boston, MA).

### Larvae collection and growth rate

Two thousand third instar larvae were collected near the experimental site in late May 2010 for use in this experiment. At the beginning of the experiment, visible predaceous invertebrates and phytophagous insects on aboveground plants in the cages were eliminated by hand. According to the field experiment of Yan et al. (2006), 90 third instar larvae were weighed and placed at the center of each cage on 2 June 2010.

Fifteen larvae were randomly removed, weighed, and returned to each cage until the larvae were near the end of their seventh (i.e., final) instar. Larvae were sampled about once a week. The larval growth trajectory was exponential during the growing season of 2010 (larval body mass = 0.004e^0.053D^, *r*^2^ = 0.922, *n *=* *160, *P *<* *0.01). The equation used to calculate relative growth rate (RGR) was *RGR* = ln (*M2*/*M1*)/*D*, where *M2* is the final weight, *M1* the initial weight, and *D* is development time in days.

### Pupal weight, pupal density, and potential fecundity

Larvae of *G. menyuanensis* pupated under leaves of broadleaf weeds (i.e., *S. superba*,*G. straminea,* and *L. tibetica*). Pupae with cocoons in cages were observed and marked at 2-day intervals from mid-July to late August and were sexed by the appearance and size of cocoons. Healthy pupae were weighed 1 day after pupation and put back into the cages. Total pupal amounts in cages were counted and used to calculate pupal density.

A nondestructive sampling method was conducted to estimate the egg load of *G. menyuanensis*. In August, female pupae were checked and marked in a meadow near the experimental site that was synchronous with the controlled experiment. Pupae with cocoons were weighed and put back 1 day after pupation. Marked cocoons with eggs were collected from the field on the tenth day after mating. Eggs were removed from cocoons and counted in the laboratory. The equation used to simulate potential fecundity (PF) was *PF* = 31.680 + 0.583*W* (*r*^2^ = 0.71, *n *=* *120, *P *<* *0.01), where *W* is female pupal weight. Potential fecundity was estimated based on female pupal weight under different treatments.

### Overwintering survival rate of first instar larvae

Two thousand first instar larvae were collected near the experimental site in October 2010. Two 60-mesh white nylon screen cages (10 cm in diameter and 20 cm long) were placed in each plot. Fifty larvae were put in each cage. Neonate larvae congregated in bushy litter of graminoids for overwintering. The healthy larvae were checked and counted in the early April 2011.

### Changes in aboveground biomass and graminoid coverage induced by grassland caterpillar

In each plot, changes of aboveground biomass and graminoid coverage induced by *G. menyuanensis* were the differences between a 100 × 100 cm quadrat without grassland caterpillars and a 40 × 40 cm cage with grassland caterpillars. A nondestructive sampling method was described in detail by Wang et al. (2012) to estimate aboveground biomass. The equation used to simulate aboveground biomass (AGB) was *AGB* = −5.7575 + 0.0839*C* + 5.6656*H* (*r*^2^ = 0.84, *n *=* *210, *P *<* *0.001), where *C* is the total sward coverage and *H* is mean sward height. The mean height and mean coverage were measured using the 100 × 100 cm quadrat divided into 400 squares (5 × 5 cm) and the 40 × 40 cm cage divided into 100 squares (4 × 4 cm). Mean coverage of different species was calculated as the total coverage in squares with species presence divided by the total number squares. Graminoid coverage was calculated as the sum of mean coverage of each graminoid species.

### Data analysis

GLMs followed by multicomparisons using Duncan's test were applied to test the effects of warming, grazing, and their interactions on all measured variables. Multiple stepwise regression analysis was conducted to test the relationships between growth rate or pupal weight and sward height, sward coverage, plant litter mass, and N concentration of graminoids. Statistical analyses were performed with SAS 9.2 (SAS Institute Inc., Cary, NC). All significant differences were at *P *≤* *0.05.

## Results

### Sward structure and nitrogen concentration of graminoids

Experimental warming and grazing altered sward structure (i.e., sward height, sward coverage, and plant litter mass). Grazing significantly decreased sward height in 2010 (*P *<* *0.001), sward coverage in 2010 (*P *=* *0.002), and plant litter mass in 2009 (*P *<* *0.001) (Tables2). Interaction between warming and grazing on plant litter mass was also present (*P *=* *0.004) (Table1). The plant litter mass under the WG treatment was lower than that predicted from the sum of the individual effects of warming and grazing. Grazing significantly increased total N concentration in fully mature leaves of graminoids in August 2010 (*P *<* *0.001), whereas warming decreased the total N concentration (*P *=* *0.068) (Tables2).

**Table 1:** Summary of GLMs for the effects of warming (W) and grazing (G) on sward height, sward coverage, plant litter mass, nitrogen (N) concentration in graminoids, larval growth rate, pupal weight, potential fecundity, overwintering survival rate, pupal density, and reductions induced by *Gynaephora menyuanensis* in aboveground biomass (AGB) and graminoid cover

Index	Source	df	*F*	*P*
Sward height	W	1,12	3.26	0.096
	G	1,12	49.33	<0.001*
	W × G	1,12	0.64	0.438
Sward coverage	W	1,12	0.95	0.348
	G	1,12	15.86	0.002*
	W × G	1,12	0.11	0.751
Plant litter mass	W	1,12	1.06	0.323
	G	1,12	35.61	<0.001*
	W × G	1,12	12.17	0.004*
N in graminoids	W	1,12	4.01	0.068
	G	1,12	60.13	<0.001*
	W × G	1,12	0.53	0.483
Larval growth rate	W	1,12	2.76	0.122
	G	1,12	25.38	<0.001*
	W × G	1,12	2.71	0.126
Pupal weight	Sex	1,155	343.92	<0.001*
	W	1,155	2.10	0.149
	G	1,155	33.29	<0.001*
	W × G	1,155	0.001	0.983
Potential fecundity	W	1,77	2.33	0.131
	G	1,77	21.06	<0.001*
	W × G	1,77	0.01	0.931
Overwintering survival rate	W	1,12	0.001	0.977
	G	1,12	2.27	0.158
	W × G	1,12	0.20	0.666
Pupal density	W	1,12	0.97	0.345
	G	1,12	33.51	<0.001*
	W × G	1,12	1.44	0.254
Reduction in AGB	W	1,12	2.48	0.142
	G	1,12	163.30	<0.001*
	W × G	1,12	7.33	0.019*
Reduction in graminoid cover	W	1,12	0.42	0.530
	G	1,12	91.35	<0.001*
	W × G	1,12	2.83	0.119

* *P *≤* *0.05.

**Table 2:** Sward height, sward coverage, plant litter mass, and nitrogen (N) concentration in graminoids under different treatments

Item	Treatment			
	NWNG	NWG	WNG	WG
Sward height (cm)	8.1 a	5.0 b	9.4 a	5.5 b
Sward coverage (%)	167.0 ab	132.3 c	172.3 a	142.8 bc
Plant litter mass (g/m^2^)	127.6 b	102.1 bc	174.3 a	76.7 c
N in graminoids (%)	2.0 b	2.4 a	1.8 b	2.3 a

NWNG, no warming with no grazing; WNG, warming with no grazing; NWG, no warming with grazing; WG, warming with grazing.

Letters in the same row indicate significant difference at *P *≤* *0.05 in descending order.

### Larval growth rate and pupal weight

**Figure 1:**
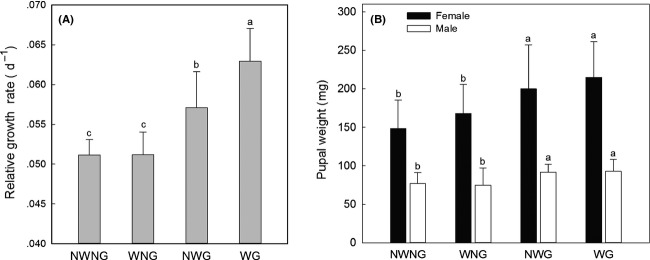
Larval growth rate (A) and pupal weight (B) under different treatments in 2010. NWNG: no warming with no grazing; WNG: warming with no grazing; NWG: no warming with grazing; WG: warming with grazing. Letters indicate significant difference at *P *≤* *0.05 in descending order.

Grazing significantly increased larval growth rate from 2 June to 24 July (*P *<* *0.001) and pupal weight (*P *<* *0.001) in 2010 (Table1, Fig.1). The increase in larval growth rate under WG (23%) was significantly higher than that under NWG (12%) (Fig.1A). Male pupae were significantly smaller than the females (*P *<* *0.001) (Table1, Fig.1B). Female pupal weight increased by 35% under NWG and 45% under WG, while male pupal weight increased by only 20% under NWG and 22% under WG, compared to NWNG (Fig.1B). The increase in female pupal weight under WG was higher by 10% than that under NWG (Fig.1B).

### Relationships between grassland caterpillar performance and plant factors

Larval growth rate was significantly negatively correlated with plant litter mass. Plant litter mass explained 54% of the variation in larval growth rate (Table3, M1). Female pupal weight was significantly positively correlated with N concentration in graminoids. Nitrogen concentration in graminoids explained 75% of the variation in female pupal weight (Table3, M2). There were two effective linear models on the relationship between male pupal weight and plant factors (Table3, M3, 4). With a higher influence in M4, plant litter mass and N concentration in graminoids explained 93% of the variation in male pupal weight. Male pupal weight was significantly negatively correlated with plant litter mass, but positively correlated with N concentration in graminoids.

**Table 3:** Models of larval growth rate (GT), female pupal weight (FPW), male pupal weight (MPW), sward height (SH), sward cover (SC), plant litter mass (PLM), and nitrogen (N) concentration in graminoids through stepwise regression

Dependent variable	Independent variables	Linear model	*R* ^2^	*P*
GT	SH, SC, PLM	M1: GT = 0.0685 − 0.0001PLM	0.54	0.01
FPW	SH, SC, PLM, N	M2: FPW = −0.736 + 85.870N	0.75	<0.001
MPW	SH, SC, PLM, N	M3: MPW = 6.889 + 35.637N	0.85	<0.001
		M4: MPW act = 56.495 − 0.133PLM + 19.926N	0.93	<0.001

### Potential fecundity, overwintering survival rate, and pupal density

**Figure 2:**
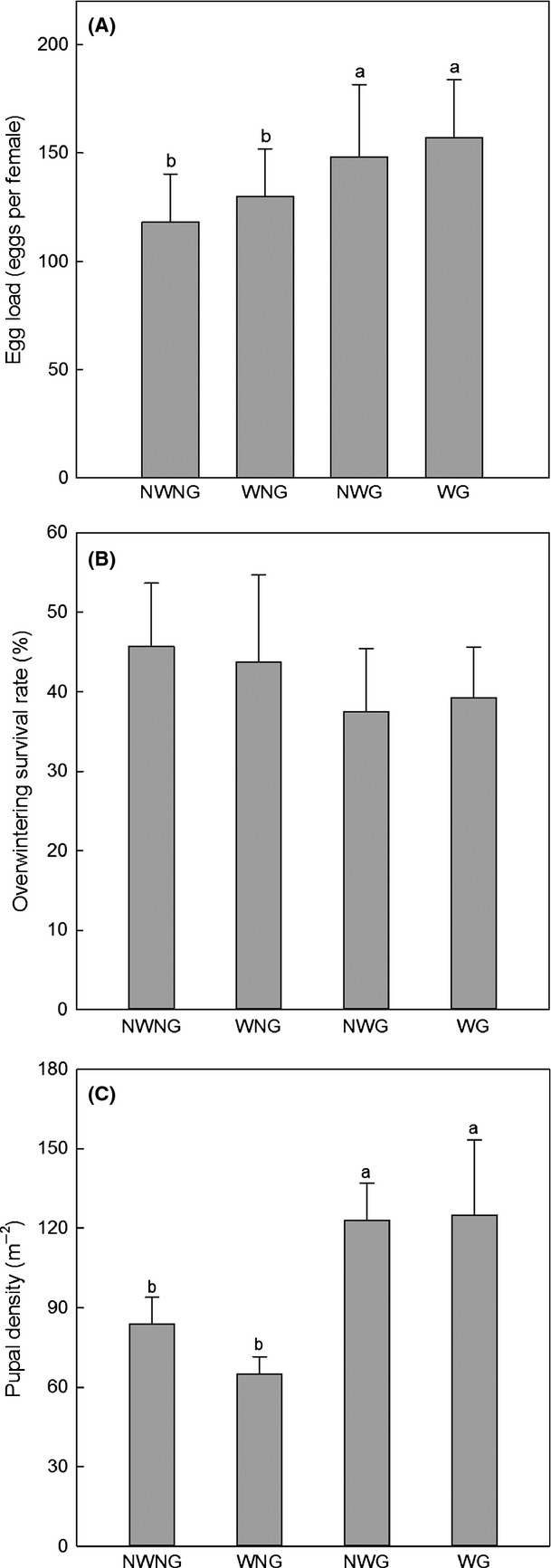
Potential fecundity in 2010 (A), overwintering survival rate during the winter of 2010 (B), and pupal density in 2011 (C) under different treatments. NWNG: no warming with no grazing; WNG: warming with no grazing; NWG: no warming with grazing; WG: warming with grazing. Letters indicate significant difference at *P *≤* *0.05 in descending order.

Grazing significantly increased potential fecundity in 2010 (*P *<* *0.001) (Table1, Fig.2A). The increase in potential fecundity under WG (33%) was higher than that under NWG (25%) (Fig.2A). There were no significant effects of warming, grazing, and their interaction on the overwintering survival rate of first instar larvae from November 2010 to April 2011 (Table1, Fig.2B). Grazing significantly increased pupal density in 2011 (*P *<* *0.001) (Table1, Fig.2C). Pupal density significantly increased by 43% under NWG and 49% under WG, respectively, while pupal density decreased by 22% under WNG, compared to NWNG (Fig.2C).

### Reductions in aboveground biomass and graminoid coverage induced by grassland caterpillar

**Figure 3:**
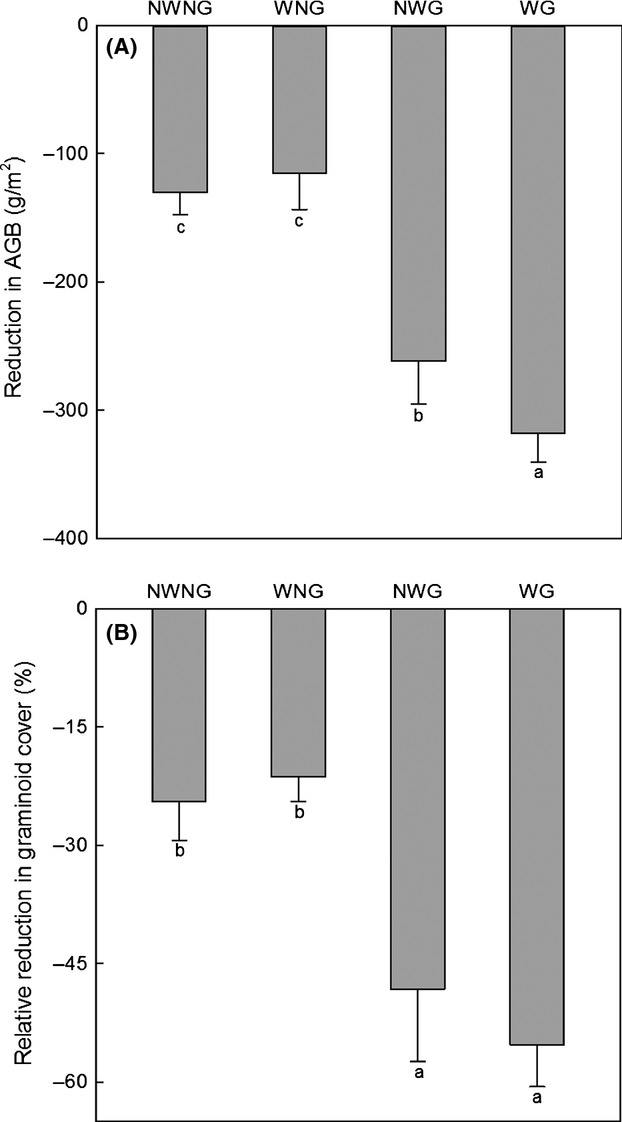
*Gynaephora menyuanensis* induced reductions in aboveground biomass (AGB) (A) and graminoid cover (B) under different treatments in 2011. NWNG: no warming with no grazing; WNG: warming with no grazing; NWG: no warming with grazing; WG: warming with grazing. Letters indicate significant difference at *P *≤* *0.05 in descending order.

Grazing significantly enhanced the negative effects of *G. menyuanensis* on aboveground biomass (*P *<* *0.001) and graminoid coverage (*P *<* *0.001) in 2011 (Table1, Fig.3). Interaction between warming and grazing on the reduction in aboveground biomass was also present (*P *=* *0.019) (Table1). In the presence of grazing, warming significantly increased the reduction in aboveground biomass, whereas warming decreased that in the absence of grazing (Fig.3A). The increase in relative reduction in graminoid coverage under WG (31%) was higher than that under NWG (24%) (Fig.3B).

## Discussion

### Effects of warming and grazing on grassland caterpillar

As poikilotherms, insects are able to achieve and maintain specific body temperatures through behavioral thermoregulation such as basking and shade seeking (Heinrich 1996). The regulated body temperature often coincides with the optimum of one or more physiologically important processes, such as feeding rate (Lactin and Johnson 1995), feeding efficiency (Porter 1982), growth rate (Knapp and Casey 1986), and/or metabolic efficiency (Kukal et al. 1988; Bennett et al. 1999). Caterpillars of high arctic *Gynaephora* moths (*Gynaephora rossii* Curtis and *Gynaephora groenlandica* Wöcke) bask to elevate their body temperatures to ∼10–20°C above ambient temperature (Kevan et al. 1982; Kukal et al. 1988). The abiotic and biotic environments of the TP are similar to those of the arctic tundra, with long severe winters and short cool summers (Zhao and Zhou 1999). Like other congeneric species, *G. menyuanensis* black woolly bear caterpillars also bask by grasping at the top of grass leaves after sunrise (Yan et al. 2006). Microhabitat structure plays an important role in thermoregulation (Willott 1997; Nice and Fordyce 2006; Ashton et al. 2009). The availability of light for basking is highly dependent on the degree of shading by vegetation. In our study, grazing increased the sward openness with a lower plant litter mass, which was beneficial to *G. menyuanensis* performance. The result suggests that basking is an important thermoregulatory strategy to grassland caterpillars in the high-cold meadows. Therefore, the effect of sward structure on behavioral thermoregulation ability should be considered when assessing the performance of *G. menyuanensis* under future warming with grazing conditions.

Nitrogen, which plays a key role in protein metabolism, is a limiting nutrient for herbivores, due to its low concentration in forage tissues relative to herbivore requirements (White 1993). Plant N content is closely related to the performance and dynamics of herbivorous insects (Mattson 1980). In our study, grazing significantly increased N concentration of graminoids, which was significantly positively correlated with the body size of *G. menyuanensis*. The result suggests that an increase in N content of herbaceous food sources under grazing may enhance *G. menyuanensis* performance by relieving nitrogen limitation.

The pupae of *G. menyuanensis* exhibit sexual size dimorphism. The body size of the male, which has six instars during the larval period, is much smaller than that of the female, which has seven instars (Yan et al. 2006). In this study, increases in female pupal weight were about twofold greater than that of the male under grazing treatments. One possible explanation is that the larger variation in female pupal weight was associated with the longer larval period (∼2 weeks). Attaining a higher pupal weight is critical due to a strong correlation between female body size and fecundity (Tammaru et al. 1996). Larger females of *G. menyuanensis* laid more eggs than smaller females. There was higher potential fecundity of *G. menyuanensis* under grazing, which would increase caterpillar density of next year in the meadows.

Neonate larvae of *G. menyuanensis* congregate in bushy litter of graminoids for overwintering in diapause. Mean temperature was −8.3°C from November 2010 to April 2011, with an extreme minimum temperature at about −30°C. There was no significant effect of warming on larval overwintering survival rate in 2010, suggesting that the first instar larvae of *G. menyuanensis* in diapause are not sensitive to the increase of ambient temperature (1.5/2.0°C, daytime/nighttime) during long severe winter due to low background temperature.

### The feedback of grassland caterpillar to plants

The basic characteristic of degraded alpine meadow is a reduction of graminoids, which decreases aboveground biomass in the region (Zhou et al. 2005; Zhao 2009). Several studies indicate that aboveground biomass has variable responses to warming in arctic and alpine regions, with reported increases, decreases or no change (Arft et al. 1999; Rustad et al. 2001; Wan et al. 2005; Klein et al. 2007; Post and Pedersen 2008). Under our free-air temperature enhancement system, warming significantly increased aboveground biomass, whereas grazing reduced the response of aboveground biomass to warming (Wang et al. 2012). Similar to meta-analysis of 13 sites (Arft et al. 1999), we observed that warming increased graminoid coverage, but grazing decreased it (Wang et al. 2012). *Gynaephora menyuanensis* is the major pest in the northern TP and devours graminoid leaves in alpine meadows (Yan et al. 1995). In our study, grazing significantly enhanced the negative effects of *G. menyuanensis* on aboveground biomass and graminoid coverage. Therefore, *G. menyuanensis* reduced the response of aboveground biomass and graminoid coverage to warming and significantly enhanced the reductions in aboveground biomass and graminoid coverage induced by sheep grazing. Our findings suggest that *G. menyuanensis* damage intensifies degradation of alpine meadow under sheep grazing.

In our study, the positive effects of grazing on *G. menyuanensis* performance and its damage were exacerbated by the warming treatment. The result suggests that the fitness of *G. menyuanensis* would increase under future warming with grazing conditions, thereby posing a greater risk to the health of alpine meadow and livestock. This reinforces the critical need for more detailed monitoring and intensified management strategies to both grassland caterpillars and livestock under future climate change.
